# The relationship between different severity of COVID-19 pneumonia and arterial stiffness based on artificial intelligence analysis

**DOI:** 10.3389/fmed.2025.1594570

**Published:** 2025-08-29

**Authors:** Lingheng Wu, Lin Jin, Xinyi Li, Mengjiao Zhang, Jianxiong Chen, Xiaobo Tang, Lianfang Du, Xifu Wang, Zhaojun Li, Xianghong Luo

**Affiliations:** ^1^Department of Ultrasound, Mindong Hospital Affiliated to Fujian Medical University, Ningde, China; ^2^Department of Ultrasound, Shanghai General Hospital of Nanjing Medical University, Shanghai, China; ^3^Department of Ultrasound, Guanghua Hospital Affiliated to Shanghai University of Traditional Chinese Medicine, Shanghai, China; ^4^Department of Ultrasound, Shanghai General Hospital, Shanghai Jiaotong University School of Medicine, Shanghai, China; ^5^Business School, Hubei University, Wuhan, China; ^6^Department of Medical Imaging, Weifang Medical University, Weifang, China; ^7^Department of Radiology, Jiading Branch of Shanghai General Hospital, Shanghai Jiaotong University School of Medicine, Shanghai, China; ^8^Department of Echocardiography, Shanghai General Hospital, Shanghai Jiaotong University School of Medicine, Shanghai, China

**Keywords:** COVID-19, arterial stiffness, arterial velocity pulse index, lung involvement, artificial intelligence

## Abstract

**Rationale and objectives:**

This study aimed to investigate the correlation between the severity of pulmonary infection and arterial stiffness pulse in coronavirus disease 2019 (COVID-19) patients using artificial intelligence (AI) quantitative analysis.

**Materials and methods:**

A total of 100 COVID-19 patients (with a mean age of 76 years) were enrolled in this study and were stratified into three groups based on the severity of their condition: mild, moderate, and severe. An AI imaging diagnostic system was used for automatic identification and quantitative analysis of infected lesions. Arterial stiffness was evaluated using the arterial velocity pulse index (AVI). Multiple linear regression analyses were performed to investigate the independent associations between the AVI, inflammatory markers, and radiographic parameters. Hazard ratios and Kaplan–Meier curves were produced to assess the association between arterial stiffness and radiographic parameters in relation to the composite outcome of all-cause mortality.

**Results:**

The AVI was elevated in the moderate and severe groups compared to the mild COVID-19 group (*p* < 0.001). Multiple linear regression analyses showed that the AVI was associated with the highest percentage of lobe infection (*β* = 0.813, 95%CI, 0.056–0.394, *p* = 0.011). Multivariable Cox regression showed that an AVI ≥ 33 was associated with all-cause mortality {hazard ratio, 16.201 [95% confidence interval (CI), 1.601, 163.987]}.

**Conclusion:**

As the severity of pneumonia infection increased in COVID-19 patients, vascular endothelial function was impaired, leading to increased arterial stiffness. The AVI was associated with the highest percentage of lobe infection, and the severity of pneumonia was identified as an independent risk factor for increased arterial stiffness. Worsening arterial stiffness poses an increased risk of death in COVID-19 patients.

## 1 Introduction

In recent years, coronavirus disease 2019 (COVID-19) has posed a significant challenge for healthcare systems worldwide (1). The primary complications of COVID-19 are viral pneumonia and acute respiratory distress syndrome (2). However, there is growing evidence that COVID-19 is also associated with cardiovascular system complications, such as microvascular thrombosis, venous thromboembolism, and arterial thrombosis, which may contribute to the high rates of morbidity and mortality in COVID-19 patients (3, 4). Importantly, the interactions between COVID-19 and the cardiovascular system are multifaceted, extending from direct or inflammatory injury to the myocardium to endothelial involvement and impairment (5, 6). Furthermore, studies have shown that arterial stiffness is significantly higher in patients during the acute phase of COVID-19 (7, 8).

Clinical studies have shown that arterial stiffness increases as a result of the progressive loss of arterial elasticity, and it is accelerated by conditions that increase cardiovascular risk (9). Notably, increased arterial stiffness is a significant risk factor for many cardiovascular diseases (such as heart failure and myocardial infarction) (10). The arterial velocity pulse index (AVI) is a non-invasive validation method for assessing arterial stiffness (11, 12). It is an indirect method of obtaining central arterial stiffness by analyzing the proximal brachial artery pressure waveform using an oscillometric transducer, which expands the systolic pressure in the upper arm blood pressure cuff. The waveform is then analyzed to determine central arterial stiffness. An increased AVI value indicates increased arterial stiffness from the center to the periphery (13).

Chest computed tomography (CT) is a sensitive imaging method used for evaluation and follow-up of COVID-19 pneumonia (14). However, currently, CT scans for COVID-19 are often manually evaluated by radiologists, who are unable to accurately and quantitatively evaluate disease severity (15). Recently, artificial intelligence (AI)-based algorithms have been used for the quantitative evaluation of CT changes and assessment of disease severity for COVID-19 pneumonia (16, 17). A deep learning (DL)-based segmentation system has been developed to automatically segment and quantify infection regions in CT scans of COVID-19 patients. This system is highly accurate in automatic infection delineation (an average Dice similarity coefficient of 91.6% between automatic and manual segmentations), percentage of infection (a mean estimation error of 0.3 for the whole lung), and severity prediction (73.4%) (18). However, the relationship between arterial stiffness and the quantitative CT parameters of COVID-19 pneumonia remains unclear.

In this study, we aimed to quantitatively assess the initial CT findings of COVID-19 pneumonia in different severity groups and to investigate the relationship between quantitative CT parameters and arterial stiffness.

## 2 Materials and methods

### 2.1 Study population

The present single-center, prospective observational cohort study was conducted between December 2022 and January 2023. Subjects diagnosed with COVID-19 who had complete lung CT data and blood test results were included. Subjects with the following conditions were excluded from the study: other infectious pneumonias, severe mental illness, atrial fibrillation, a history of myocardial infarction, severe renal insufficiency, severe valvular heart disease, and pregnancy. Additionally, individuals unable to obtain an AVI due to upper limb infections, previous vascular interventions, or amputations were excluded. All patients provided informed consent, and approval from the ethics committee (2024TG001) was obtained. The study adhered to the guidelines of the Declaration of Helsinki.

### 2.2 Definition of COVID-19 infection

Patients were stratified into three groups—mild, moderate, and severe—according to the “Diagnosis and Treatment Protocol for Novel Coronavirus Pneumonia (Trial Version 10)” by the National Health Commission of China (19). Mild: This group includes individuals with upper respiratory tract infections, presenting symptoms such as dry throat, sore throat, cough, and fever. Moderate: Patients in this group have a persistent high fever for more than 3 days and/or experience symptoms such as cough and shortness of breath; however, their respiratory rate (RR) remains below 30 breaths/min, and their oxygen saturation is above 93% while resting on room air. Imaging shows a characteristic COVID-19 infection with pneumonia. Severe: Adults with any of the following that cannot be explained by other reasons besides the new coronavirus infection are included: (1) shortness of breath with an RR ≥ 30 beats/min, (2) oxygen saturation ≤ 93% on air inhalation at rest, (3) partial pressure of arterial oxygen (PaO2)/oxygen uptake (FiO2) ≤ 300 mmHg (1 mmHg = 0.133 kPa), with correction for PaO2/FiO2 according to the following formula in high altitude areas8 (more than 1,000 m): PaO2/FiO2 × [760/atmospheric pressure (mmHg)]. The PaO2/FiO2 should be corrected according to the following formula: PaO2/FiO2 × [760/atmospheric pressure (mmHg)]. (4) There is a progressive exacerbation of clinical symptoms, with a marked increase in the size of the lung lesion by >50% within 24–48 h on imaging. These criteria are used to categorize patients into mild, moderate, and severe groups.

### 2.3 Baseline clinical and laboratory characteristics

All patients underwent a physical examination, and basic data were recorded after admission. Gender, age, smoking, height, weight, medical history, hypertension, diabetes, coronary artery disease, cerebrovascular disease, chronic renal disease, and pulse oxygen saturation (SpO_2_) were recorded. Body mass index (BMI) was calculated as weight (kg)/height (m^2^).

Laboratory findings included leukocyte count, neutrophil percentage, lymphocyte percentage, C-reactive protein (CRP), and interleukin 6 (IL-6). The neutrophil-to-lymphocyte ratio (N/L ratio) was defined as the ratio of the neutrophil count to the lymphocyte count.

To exclude superadded bacterial infections, all enrolled patients underwent sputum testing and blood cultures within 48 h of hospital admission. Patients with clinically significant bacterial growth were excluded from this study.

Finally, the study also recorded the length of hospital stay and 30-day mortality rates.

### 2.4 AVI measurement

AVI measurements were performed on all patients on the same day as their laboratory examinations. The AVI was measured by the cuff oscillation method using PASESA AVE-2000Pro (PASESA AVE-2000, DAIWA Healthcare, Shenzhen, China). Patients rested for at least 5 min before measurement, and avoided smoking and caffeinated beverages for at least 24 h before the measurement. The AVI, systolic blood pressure (SBP), diastolic blood pressure (DBP), and heart rate (HR) were measured with the patient in a seated position with a cuff around the left upper arm.

### 2.5 Acquisition of CT images and image analysis

CT examinations were performed on all patients on the same day after the AVI measurements. Non-contrast-enhanced CT examinations were acquired using a multidetector CT scanner with 64 detector rows (uCT 760, United Imaging, Shanghai, China). The scanning parameters are as follows: tube voltage of 120 kV, automatic milliamp tube current, collimation width of 40 mm, screw pitch of 0.984, rotation time of 0.6 s, layer thickness of 5 mm, and matrix of 512 × 512. The patients were scanned in the supine position, and the scanning began after deep inspiration and breath-holding, without the use of contrast. The scan covered the area from the apex to the base of the lung.

An AI imaging diagnostic system (uAI Discover—Pneumonia, United Imaging Intelligence, Shanghai, China) was used for the automatic identification and quantitative analysis of infected lesions. A previous study showed that the system achieved high accuracy in automatically delineating lesion regions (with an average dice similarity coefficient of 91.6%) and lesion percentage metrics (with a sensitivity of 99.76% and a misdiagnosis rate of only 0.08%) compared to 95.3% for delineation (20).

**Figure 1:**
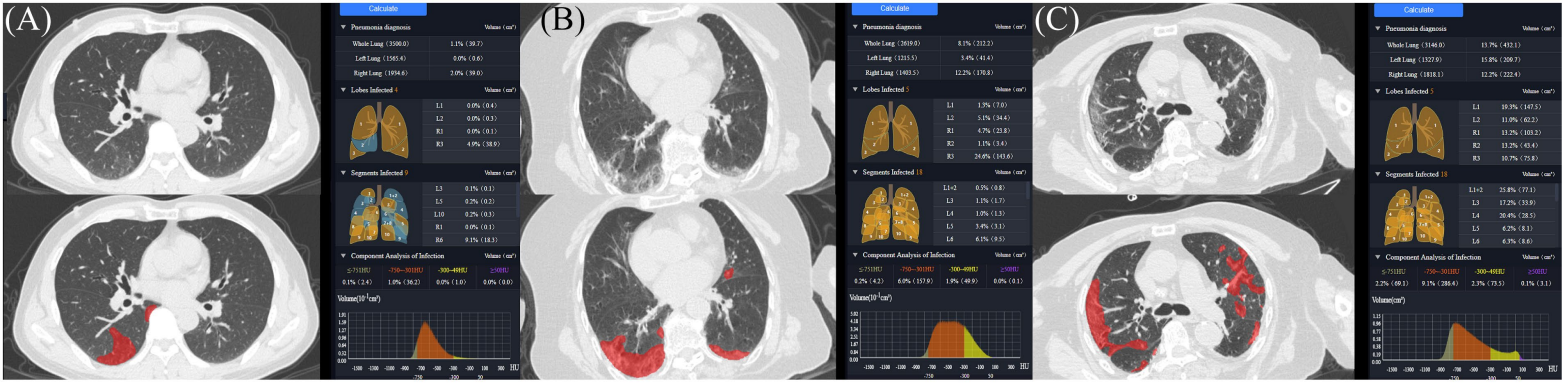
AI-assisted automatic lesion extraction and quantitative analysis. **(A)** 49-year-old man with mild symptoms of COVID-19, whole lung, highest lobe, highest segmental infection volume and percentage at the threshold of −300 are 39.7 cm^3^, 38.9 cm^3^, 18.3 cm^3^, 1.1, 4.9, 9.1%, respectively; **(B)** An 86-year-old woman with moderate symptoms of COVID-19, whole lung, highest lobe, highest segmental infection volume and percentage at the threshold of −300 HU are 212.2 cm^3^, 34.4 cm^3^, 3.1 cm^3^, 8.1, 5.1, 3.4%, respectively. **(C)** A 69-year-old man with severe symptoms of COVID-19, highest lobe, highest segmental, infection volume and percentage at the threshold of −300 HU are 432.1 cm^3^, 147.5 cm^3^, 77.1 cm^3^, 13.7, 19.3, 25.8%, respectively.

The AI imaging system first segmented the network based on manually contoured CT data to create an initial model. Then, the initial model was applied to the next batch of infection areas, manual corrections were made, and the VB-Net model was finally built (21). A chest radiologist (with 10 years of experience) independently evaluated the CT images of all subjects without any knowledge of the patients’ signs, symptoms, or prognosis. The software automatically extracted images of the lungs (5 lung lobes and 18 lung segments) and lesion areas and automatically calculated each parameter. Parameters such as the whole lung and maximum lobe or segmental infection volume, infection percentage (the percentage of infection volume in the lung lobe segment), and the number of infected lobes or segments were obtained (Figure 1). Ground-glass opacity (GGO) is defined as a density distribution within the lesion ranging from −750 HU to −300 HU (Hounsfield units) on the CT images.

### 2.6 Outcomes

The primary outcome based on medical records is the 30-day mortality rate.

### 2.7 Repeatability

A total of 24 patients were randomly selected to assess repeatability. The AVI and the percentage of infection in the highest affected lobe percentages were measured by two doctors for inter-observer variability. One day later, one of the doctors remeasured the AVI and the percentage of infection in the highest affected lobe for intra-observer variability in these 24 patients.

### 2.8 Statistical analysis

Continuous data were described as the means and standard deviations and were compared using an ANOVA test. Non-normal variables were described as the medians and quartiles and compared using the Mann–Whitney U test. Categorical variables were described as numbers (percentages) and compared using a chi-squared test.

Because of the skewed distribution, the WBC count was log transformed to obtain normality. Spearman’s rank correlation coefficient was used to analyze the correlation between the AVI and laboratory parameters. Partial correlation was used to analyze the correlation between the AVI and age. Multiple backward linear regression analyses were used to investigate the independent associations between the AVI and inflammatory markers and radiographic parameters in patients in the moderate and severe groups, as the increase in AVI was more pronounced in these groups. The variables included in the analysis, whole lung, highest lobe, highest segmental of infection percentage, CRP, IL-6, BMI, age, and smoking status.

To study the fitted hazard ratios (HRs) and account for any confounding variables, a stepwise multivariable Cox proportional hazards analysis was conducted. An AVI ≥ 33 was considered high arterial stiffness (22), and a SpO_2_ of ≤93% was considered low oxygen saturation (19). The following covariates were identified as most influential ones: age, male gender, heart rate, SpO_2_ ≤ 93, N/L ratio, CRP > 8 mg/L, AVI ≥ 32, whole lung infection percentage, and highest lobe infection percentage. A log-rank test was conducted to determine the statistical significance of the Kaplan–Meier survival curves.

Bland–Altman plots were used to compare the AVI and highest lobe infection percentage measurements of the 24 subjects as mentioned above. The intraclass correlation coefficient (ICC) was used to evaluate inter- and intra-observer variabilities. All statistical analyses were performed using SPSS 23.0 (IBM, Armonk, NY, USA) statistical software. A two-tailed *p-*value of less than 0.05 was considered statistically significant.

## 3 Results

### 3.1 Baseline characteristics of the patients

A total of 100 subjects, 44 men and 56 women, aged from 18 to 80 years, with a mean age of 76 years, were included. Table 1 lists the basic data of patients and includes their clinical and laboratory characteristics.

**Table 1:** Baseline characteristics of 100 patients with COVID-19 (*n* = 100).

Variables	Mild (*n* = 15)	Moderate (*n* = 38)	Severe (*n* = 47)	*p-*value
Demographics				
Age (years)	53.2 ± 14.73	77.24 ± 9.84^*^	83.96 ± 8.12^*#^	<0.001
Male patients	3 (20.0%)	23 (60.5%)	30 (63.8%)	0.009
BMI (kg/m^2^)	23.37 ± 3.83	23.66 ± 4.25	22.81 ± 3.82	0.611
SBP (mm Hg)	125.87 ± 22.19	126.82 ± 22.42	128.00 ± 19.81	0.933
DBP (mm Hg)	83.67 ± 17.04	74.39 ± 13.13	73.96 ± 14.90	0.071
HR (beats/min)	74.87 ± 12.84	80.66 ± 11.72	88.11 ± 16.95^*^	0.005
SpO_2_ (%)	99.00 (95.75, 99.00)	96.00 (94.00, 98.50)	95.00 (91.00, 96.00) ^#^	0.016
Hypertension	7 (50.0%)	28 (73.7%)	29 (61.7%)	0.240
Diabetes	0 (0)	3 (7.9%)	3 (6.4%)	0.084
Coronary artery disease	0 (0)	3 (8.8%)	3 (6.4%)	0.633
Cerebrovascular disease	1 (33.3%)	14 (42.4%)	19 (40.4%)	0.947
Chronic kidney disease	0 (0)	5 (15.2%)	9 (19.1%)	0.510
Smoking status	0 (0)	2 (5.3%)	3 (6.4%)	0.631
Length of hospitalization (days)	10.00 (6.50, 11.00)	14.00 (10.50, 19.00)	16.50 (12.25, 21.75) ^*^	0.024
Biochemical analysis				
WBC count	0.79 ± 0.16	0.83 ± 0.20	0.95 ± 0.19^*#^	0.002
Neutrophil (%)	61.19 ± 8.83	68.26 ± 12.01	79.57 ± 10.10^*#^	<0.001
Lymphocyte (%)	29.59 ± 7.27	21.01 ± 9.68^*^	11.24 ± 6.63^*#^	<0.001
N/L ratio	1.96 (1.54, 2.60)	3.23 (2.27, 5.46)	7.88 (4.98, 14.43) ^*#^	<0.001
Inflammatory markers				
C-reactive protein (μg/L)	0.00 (0.00, 4.48)	5.58 (2.45, 15.25)	18.30 (5.28, 60.16) ^*#^	<0.001
IL-6 (pg/mL)	2.24 (0.00, 12.12)	10.52 (3.69, 31.99)	40.58 (10.57, 94.18) ^*^	0.009
Outcome				
Deceased	0 (0)	0 (0)	16 (34) ^*^	<0.001
Arterial stiffness indices				
AVI	15.13 ± 3.93	21.79 ± 5.07^*^	21.06 ± 6.45^*^	0.001

Data are presented as the mean (SD) or median (IQR) for continuous variables and as the number (%) for categorical variables. SBP systolic blood pressure, DBP diastolic blood pressure, BMI Body mass index; HR heart rate, SpO_2_ pulse oxygen saturation; N/L ratio, neutrophil-to-lymphocyte ratio; AVI, arterial velocity pulse index; API: arterial pressure volume index.

Compared with the mild group, ^*^p < 0.05; Compared with the Moderate group, ^#^*p* < 0.05. CRP (C-reactive protein), normal reference range <5.0 mg/L.

No significant differences were observed in smoking status, BMI, SBP, DBP, and history of hypertension among the three groups (all *p* > 0.05). Leukocyte count, neutrophil percentage, lymphocyte percentage, N/L ratio, CRP, and IL-6 levels were higher in the severe group than in the mild group (*p* < 0.05). The AVI in the moderate and severe groups was significantly higher than in the mild group [mild: 15.13 ± 3.93 (min 9; max 23) vs. moderate: 21.79 ± 5.00 (min 14; max 31) vs. severe: 21.06 ± 6.45 (min 10; max 46), *p* < 0.001].

### 3.2 Quantitative CT features

**Figure 2:**
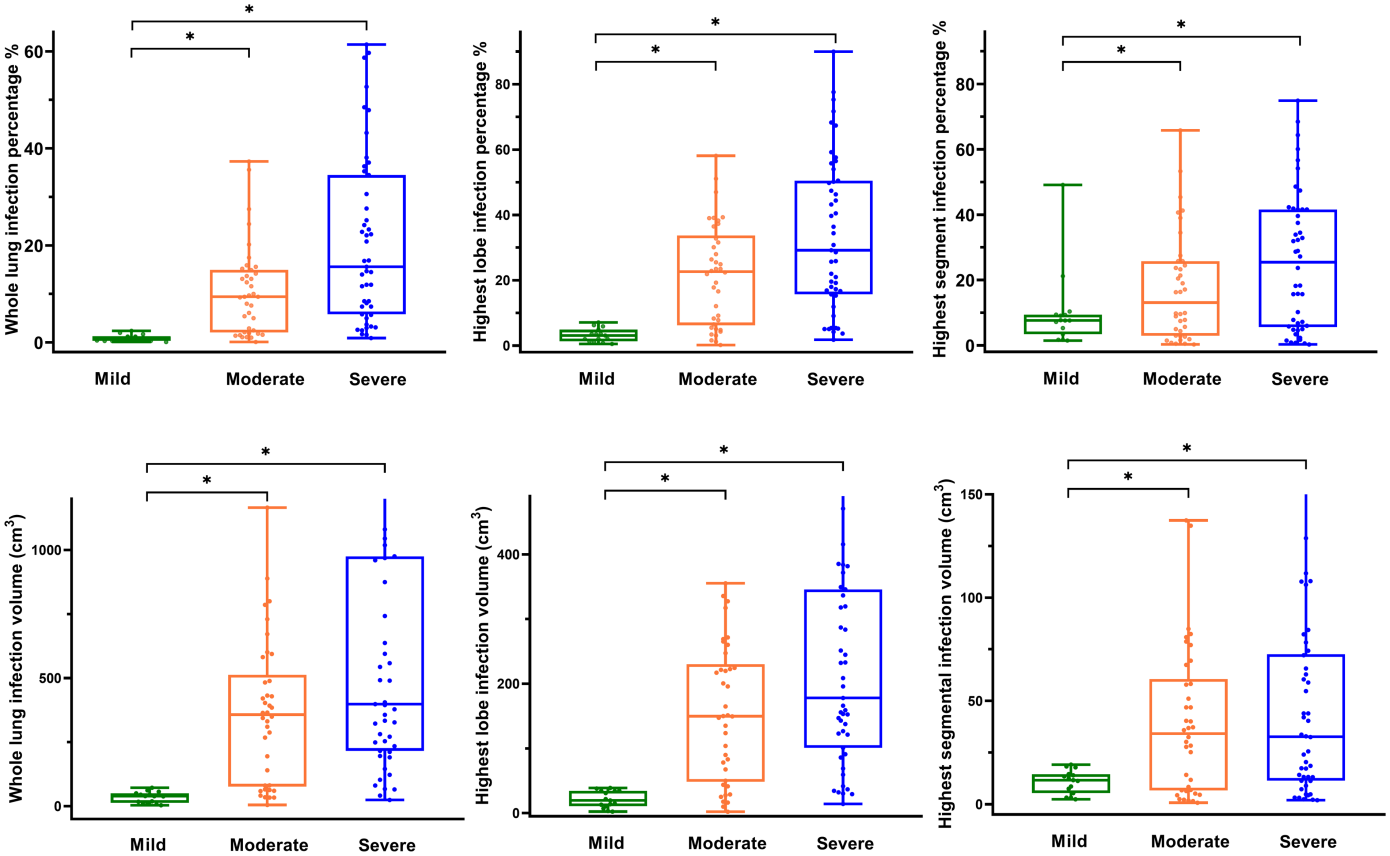
Distribution of radiographic parameters in patients with COVID-19. **(A)** Box-and-whisker plot showing whole lung infection percentage by level of COVID-19 pneumonia. **(B)** Box-and-whisker plot showing the highest lobe infection percentage by level of COVID-19 pneumonia. **(C)** Box-and-whisker plot showing the highest segment infection percentage by level of COVID-19 pneumonia. **(D)** Box-and-whisker plot showing whole lung infection volume by level of COVID-19 pneumonia. **(E)** Box-and-whisker plot showing the highest lobe infection volume by level of COVID-19 pneumonia. **(F)** Box-and-whisker plot showing the highest segmental infection volume by level of COVID-19 pneumonia.

In both the moderate and severe groups, the infection volume of the whole lung, the highest lobe, the highest segment, the GGO, the left lung, and the right lung, along with the infection percentage of the whole lung, the highest lobe, the GGO, the left lung, the right lung, and the number of infected lobes and lobe segments were higher than in the mild group (*p* < 0.05). No significant difference was observed between the moderate and severe groups (Table 2, Figure 2).

**Table 2:** Radiographic parameters of 100 patients with COVID-19.

Variables	Mild (*n* = 15)	Moderate (*n* = 38)	Severe (*n* = 47)	*P*-value
Infection volume (cm^3^)				
Whole lung	39.40 (13.00, 50.20)	357.10 (76.98, 512.43)^*^	398.40 (216.10, 975.30)^*^	<0.001
Highest lobe	19.50 (10.70, 34.40)	149.75 (48.53, 230.25)^*^	178.00 (100.80, 345.90)^*^	<0.001
Highest segment	11.70 (5.40, 14.60)	34.20 (6.70, 60.60)^*^	32.70 (11.38, 72.65)^*^	0.014
GGO	24.50 (5.60, 39.60)	220.20 (54.40, 300.88)^*^	230.60 (119.80, 584.40)^*^	<0.001
Left lung	12.50 (0.60, 19.20)	80.45 (13.83, 217.58)^*^	109.10 (29.10, 330.10)^*^	<0.001
Right lung	17.00 (8.00, 38.10)	209.75 (62.93, 351.10)^*^	298.10 (181.10, 578.10)^*^	<0.001
Infection percentage (%)				
Whole lung	1.00 (0.30, 1.20)	9.40 (2.05, 14.98)^*^	15.60 (5.80, 34.50)^*#^	<0.001
Highest lobe	3.10 (1.30, 4.90)	22.65 (6.25, 33.73)^*^	29.20 (15.70, 50.50)^*^	<0.001
Highest segment	7.60 (3.40, 9.40)	13.10 (2.95, 25.80)	25.45 (5.60, 41.60)	0.047
GGO	0.70 (0.10, 1.00)	5.25 (1.35, 9.73)^*^	8.20 (2.60, 18.10)^*^	<0.001
Left lung	0.70 (0.00, 1.10)	5.30 (1.10, 12.13)^*^	7.45 (2.35, 26.08)^*^	<0.001
Right lung	0.80 (0.50, 2.00)	10.50 (3.25, 21.40)^*^	15.20 (9.60, 36.50)^*^	<0.001
Number of infected lobe or segment				
Infected lobe	4.07 ± 0.88	4.89 ± 0.39^*^	4.89 ± 0.43^*^	<0.001
Infected segment	10.93 ± 4.50	15.97 ± 2.78^*^	16.81 ± 2.30^*^	<0.001

^*^p-value calculated for moderate vs. mild patients; ^#^*p*-value calculated for severe vs. moderate patients. GGO, ground-glass opacity.

### 3.3 Correlation analysis

In unadjusted analyses, the AVI was positively correlated with age and the number of infected lobes in all patients (*r* = 0.262, 0.026, respectively; all *p* < 0.05). After adjusting for age, this association changed such that positive correlations were observed between the AVI and the number of infected lobes and infected segments (*r* = 0.012, 0.016*, p* < 0.05, respectively) (Table 3). In a multiple linear regression involving moderate–severe patients, the AVI was significantly associated with the highest lobe infection percentage (*β* = 0.813; 95%CI, 0.056–0.394, *p* = 0.011) (Table 4).

**Table 3:** Correlation between the AVI and radiographic parameters.

Item	Correlation		Partial correlation	
	*r*	*p*-value	*r*	*p*-value
Age	0.262	0.009	–	–
Gender	0.184	0.066	0.250	0.192
Smoking status	0.180	0.074	0.356	0.058
BMI	0.008	0.936	0.111	0.565
HR	−0.073	0.473	0.105	0.586
SpO_2_	0.063	0.564	0.109	0.574
WBC count	0.005	0.957	−0.117	0.544
Neutrophil	0.124	0.228	0.158	0.412
Lymphocyte	−0.150	0.141	−0.181	0.348
N/L ratio	0.131	0.202	−0.008	0.967
C-reactive protein	0.105	0.311	−0.029	0.879
IL-6	0.295	0.085	0.078	0.687
Whole lung infection percentage	0.052	0.608	0.078	0.688
Highest lobe infection percentage	0.115	0.253	0.316	0.094
Highest segmental infection percentage	−0.128	0.208	0.078	0.686
GGO infection percentage	0.060	0.553	0.109	0.572
Left lung infection percentage	0.017	0.865	0.228	0.233
Right lung infection percentage	0.136	0.177	0.176	0.360
Number of infected lobes	0.222	0.026	0.458	0.012
Number of infected segments	0.124	0.218	0.444	0.016

HR, heart rate; N/L ratio, neutrophil-to-lymphocyte ratio; IL-6, interleukin 6; GGO, ground-glass opacity.

**Table 4:** Multiple linear regression analysis of AVI in moderate–severe cases of COVID-19 (backward).

Item	*β*	95%CI	*P*-value
Whole lung infection percentage	−0.600	−0.458–0.004	0.054
Highest lobe infection percentage	0.813	0.056–0.394	0.011

β is the regression coefficient. The following variables were included in the analysis: whole lung infection percentage, highest lobe infection percentage, highest segment infection percentage, CRP, IL-6, BMI, age, and smoking status.

### 3.4 All-cause mortality

**Figure 3:**
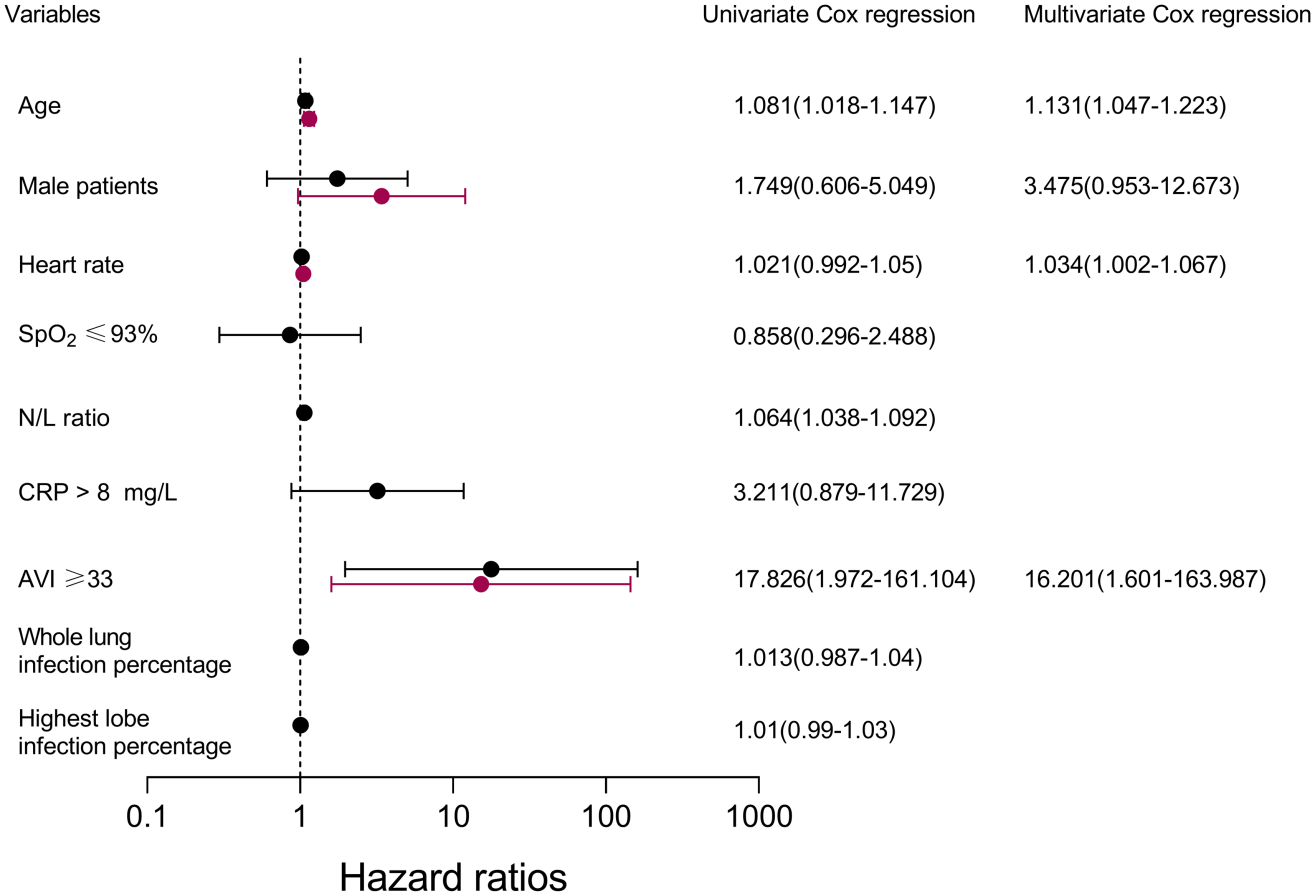
Cox proportional hazards regression: survival analysis with COVID-19. SpO_2_ pulse oxygen saturation; N/L ratio, neutrophil-to-lymphocyte ratio; AVI, arterial velocity pulse index; CRP, C-reactive protein.

The results of the Cox regression are shown in Figure 3. In the univariable Cox regression model, age, the N/L ratio, and an AVI of ≥33 remained positively associated with incident death. No significant impact was observed for heart rate, SpO_2_ ≤ 93%, CRP>8 mg/L, whole lung infection percentage, or highest lobe infection percentage. In the multivariable Cox proportional hazards analysis, an AVI ≥ 33 was independently associated with incident death (HR, 16.201; 95% CI: 1.601, 163.987; *p* < 0.001).

**Figure 4:**
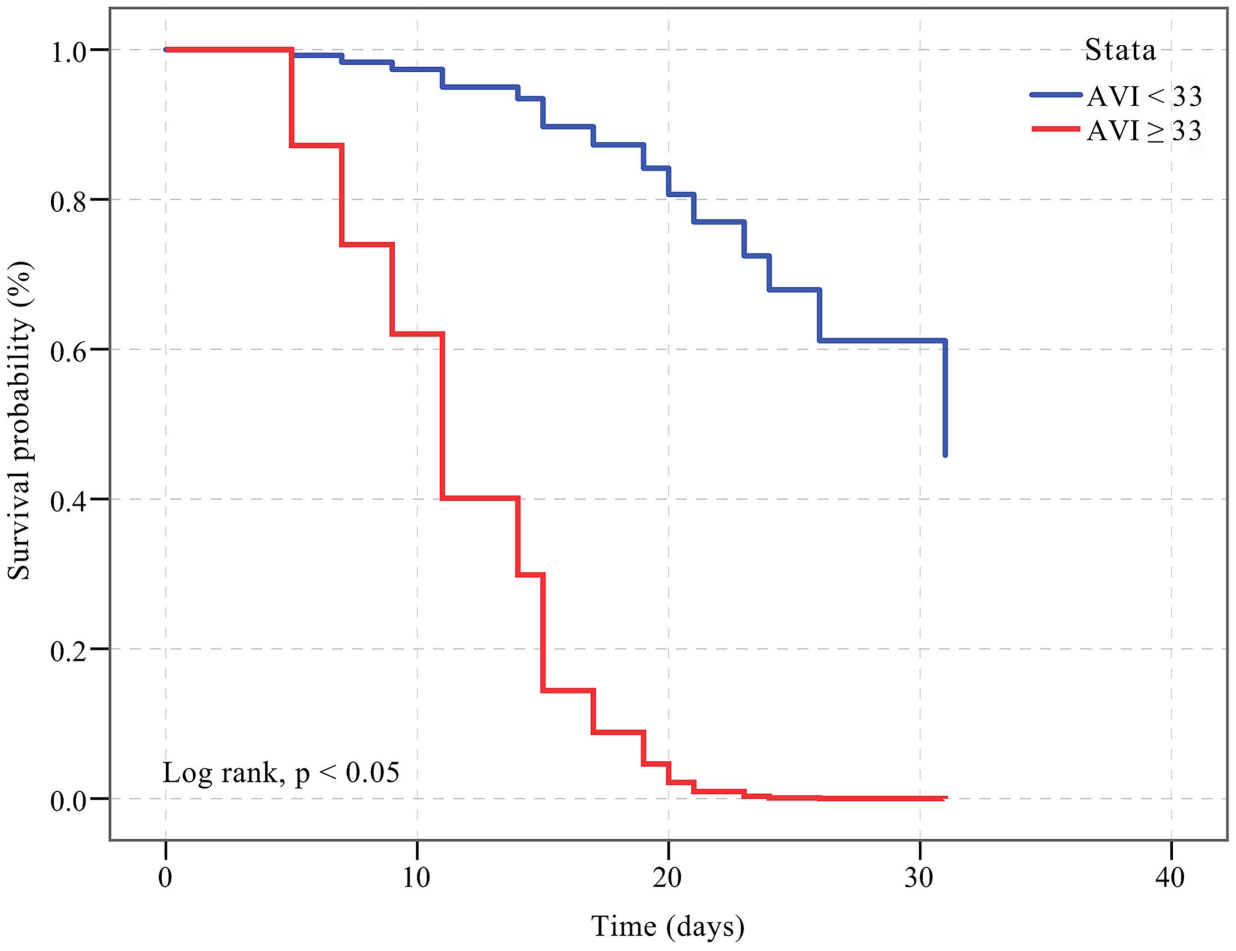
Kaplan–Meier curve showing the survival of patients with an AVI < 33 and an AVI ≥ 33. The cumulative hazard was greater for participants with an AVI ≥ 33 compared with patients with an AVI < 33 for incident death (HR, 16.201; 95% CI: 1.601, 163.987; *p* < 0.001).

The results of the Kaplan–Meier curve are shown in Figure 4. Survival was longer for patients with an AVI < 33 compared to patients with an AVI ≥ 33 (HR, 16.201; 95% CI: 1.601, 163.987; *p* < 0.001). The log-rank test indicated that this difference was statistically significant (*p* < 0.05).

### 3.5 Repeatability

**Figure 5:**
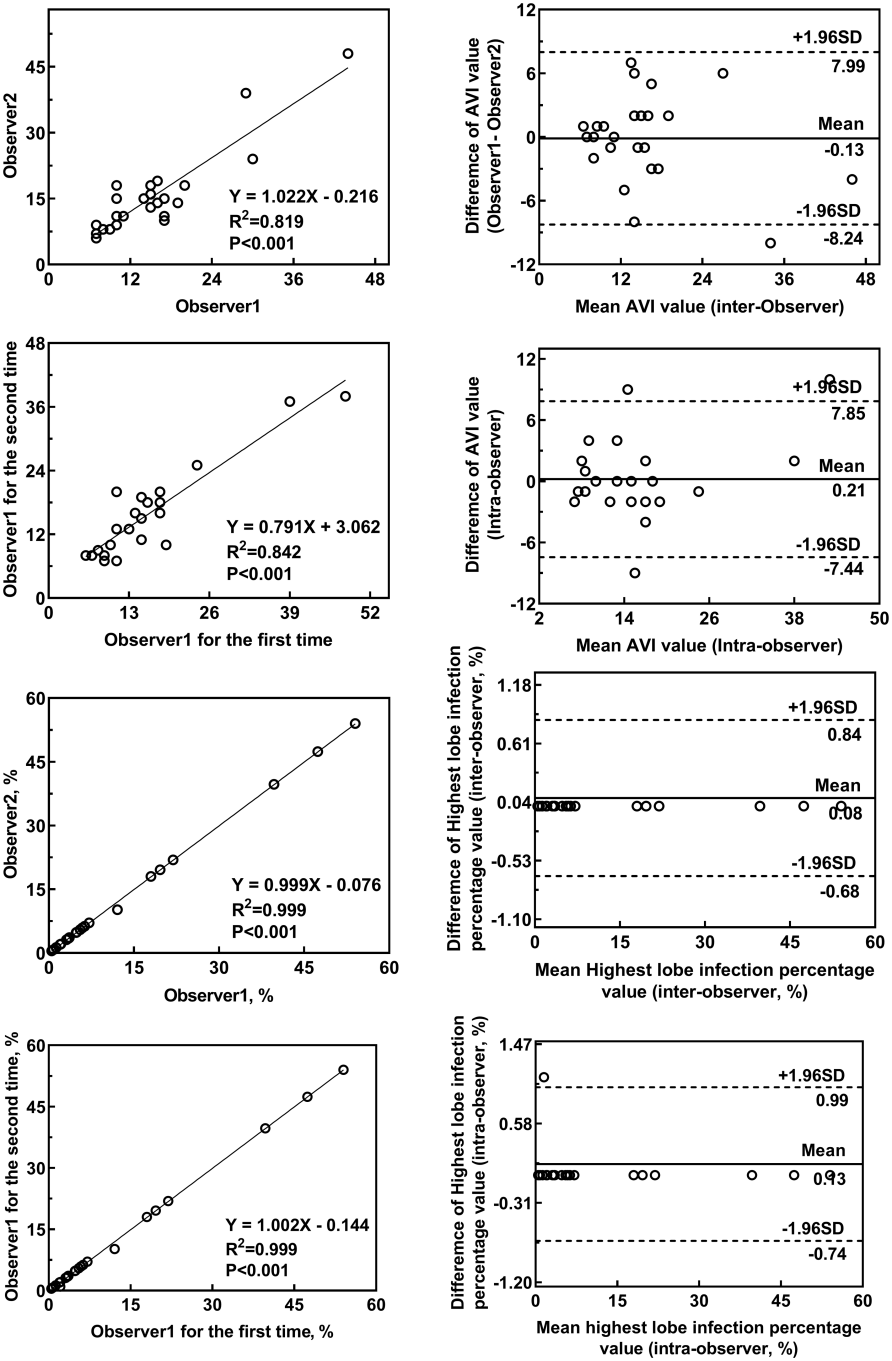
Inter-rater and intra-rater agreement for the AVI and highest lobe infection percentages. The inter-observer variability for the AVI **(A,B)** and highest lobe infection percentage **(E,F)** between the two observers showed good agreement. The intra-observer variability for the AVI **(C,D)** and highest lobe infection percentage **(G,H)** by one observer showed good agreement **(C,D)**.

Bland–Altman agreement plots for the AVI and highest lobe infection percentages are shown in Figure 5 and Table 5. The ICCs for the AVI for inter-and intra-observer reproducibility were 0.902 (95% CI, 0.787–0.956; *p* < 0.001) and 0.896 (95% CI, 0.720–0.941; *p* < 0.001), respectively. The ICCs for highest lobe infection percentages for inter-and intra-observer reproducibility were 1.000 (95% CI, 0.999–1.000; *p* < 0.001) and 1.000 (95% CI, 0.999–1.000; *p* < 0.001), respectively.

**Table 5:** Inter-observer and intra-observer reliabilities of the measurements.

Item	ICC	95%CI
AVI measured by inter-observer	0.902	0.787–0.956
AVI measured by intra-observer	0.869	0.720–0.941
Highest lobe infection percentage measured by inter-observer	1.000	0.999–1.000
Highest lobe infection percentage measured by intra-observer	1.000	0.999–1.000

ICC, interclass correlation coefficient.

## 4 Discussion

This study assessed the relationship between the severity of pulmonary infection and arterial stiffness in COVID-19 patients. According to the analysis of CT imaging by AI software, the AVI was associated with the highest lobe infection percentage. The severity of pneumonia was independently and positively associated with increased arterial stiffness and was a risk factor for it. Furthermore, an increased AVI was associated with an increased risk of worsening outcomes and could predict all-cause mortality.

Vascular function testing showed that subjects continued to have impaired vascular function parameters for 6 months after acute COVID-19, which may be related to accelerated atherosclerosis (23). The interaction between COVID-19 and arterial health involves a variety of mechanisms, including the inflammatory cascade associated with COVID-19 and the interaction between the severe acute respiratory syndrome coronavirus-2 spike protein and angiotensin-converting enzyme 2 (24, 25). In our study, an arterial stiffness index-associated AVI increased with the increasing severity of pulmonary infection. The mean AVI was 21.79 in patients with moderate infection and 21.06 in patients with severe infection, which was significantly higher than in patients with mild infection (mean AVI 15.13).

Due to age-related immune decline and decreased cardiorespiratory fitness, older adults are at a heightened risk of contracting COVID-19 (26). In our study, the age of the moderate and severe groups was higher than that of the mild group. After controlling for age bias in correlation analyses, the AVI was positively associated with the number of infected lobes and with the number of infected segments. In a recent study of a small sample of 22 COVID-19 acute patients and age-and gender-matched non-COVID-19 acute patients, it was found that both carotid-femoral pulse wave velocity and brachial-ankle pulse wave velocity were higher in patients with COVID-19 than in non-affected individuals (8). This suggests that increased arterial stiffness is independently associated with the severity of pneumonia infection.

Increased arterial stiffness is a strong risk factor for cardiovascular disease (10). An impaired AVI, as a new indicator of arterial stiffness, may indicate increased cardiac load (27). Significantly lower systemic vascular function and higher arterial stiffness caused by COVID-19 have also been revealed in young, otherwise healthy adults (28). Infection with COVID-19 was found to be strongly correlated with increased arterial stiffness (29). Previous studies have also found that an increase in arterial stiffness might be related to longer hospital stays and higher mortality rates (8). In the present study, we found that the high arterial stiffness with AVI ≥ 33 was strongly associated with an increased risk of death (HR, 16.201; 95% CI: 1.601, 163.987; *p* < 0.001). Similar studies have supported our results (30). It should be noted that in the participants, the AVI in the severe group was indeed higher, accompanied by increased vascular stiffness and elevated mortality; however, it is not a determinant of death in patients with COVID-19.

Fei Shan *et al*. (18) developed a DL-based segmentation system to automatically segment and quantify infection regions in CT scans. The system is a DL-based segmentation method that uses a “VB-Net” neural network to segment COVID-19 infection regions in CT scans, and was trained on CT scans using 249 COVID-19 patients. The results demonstrated high accuracy in automatic infection delineation and severity prediction. In our study, quantitative CT features were significantly higher in the moderate and severe groups than in the mild group, and there was better agreement.

However, the results of the present study should be interpreted with caution, since the relatively small sample size and single-center design may limit their credibility. Moreover, other types of pneumonia and the post-acute phase of COVID-19 were not considered; these will be further explored by our research group in future studies. An AI diagnostic system based on CT images was used to quantitatively analyze and automatically calculate the extent of lung infections, thus requiring larger data sets to improve AI sensitivity in the future. Moreover, an AI diagnostic system and quantitative evaluation are accurate and promising tools for detecting pneumonia in COVID-19 patients. While previous studies have found that mRNA vaccines against COVID-19 are associated with a short-term increased risk of cardiovascular events (such as myocarditis or pericarditis) (31), our future studies should consider vaccination history to better isolate disease-related cardiac changes. Our definition of high arterial stiffness is an AVI > 33 based on a large sample of the normal population. In the present study, the 30-day mortality rate in the population with an AVI > 33 was 100%. COVID-19 is characterized primarily by pulmonary failure rather than heart failure caused by vascular stiffness. This study focused on exploring the relationship between pulmonary infection and arterial stiffness, and the results provided new insights for future investigations into the AVI cutoff value for high arterial stiffness in COVID-19 patients.

## 5 Conclusion

As the degree of pneumonia infection increased in COVID-19 patients, vascular endothelial function was impaired, and arterial stiffness increased. The AVI was associated with the highest lobe infection percentage, and the severity of pneumonia was an independent risk factor for increased arterial stiffness.

## Data Availability

The raw data supporting the conclusions of this article will be made available by the authors without undue reservation.
